# Pulmonary arterial flow alterations in systemic lupus erythematosus on 4D flow CMR: a case-control study

**DOI:** 10.1186/s41747-026-00692-4

**Published:** 2026-03-04

**Authors:** Xin Chen, An Sun, Junxian Liao, Zhenhuan Wang, Xinyi Wan, Yi Xiao

**Affiliations:** Department of Radiology, Shanghai Changzheng Hospital, Shanghai, China

**Keywords:** Hemodynamics, Lupus erythematosus (systemic), Magnetic resonance angiography, Pulmonary arterial hypertension, Ventricular dysfunction (left)

## Abstract

**Objective:**

Pulmonary arterial hypertension is a severe complication of systemic lupus erythematosus (SLE). Current screening methods often miss early vascular changes. This study aimed to characterize subclinical pulmonary hemodynamic alterations in SLE patients without known pulmonary arterial hypertension using four-dimensional (4D) flow cardiovascular magnetic resonance (CMR) and to investigate their association with left ventricular diastolic function.

**Materials and methods:**

Twenty-five SLE patients without known pulmonary arterial hypertension and 25 age-matched healthy controls were enrolled. All participants underwent 3-T 4D flow CMR to quantify hemodynamic parameters, including wall shear stress (WSS), flow volume, and relative pressure in the pulmonary arteries. SLE patients were further stratified based on echocardiographic assessment of diastolic function to analyze hemodynamic coupling.

**Results:**

Compared to controls, SLE patients exhibited significantly lower maximum WSS in the main pulmonary artery (0.29 *versus* 0.33 Pa, *p* = 0.040) and asymmetric flow redistribution, characterized by higher relative pressure in the left pulmonary artery (0.54 *versus* 0.30 mmHg, *p* = 0.008) and increased flow rate in the right pulmonary artery (3.51 *versus* 2.90 L/min, *p* = 0.015). Qualitative analysis revealed vortical flow patterns in SLE patients. Subgroup analysis demonstrated that the reduction in WSS was primarily driven by patients with diastolic dysfunction (*p* = 0.006 *versus* controls).

**Conclusion:**

SLE patients without pulmonary arterial hypertension exhibit distinct subclinical pulmonary hemodynamic alterations, including lower WSS and flow asymmetry. These alterations are intimately coupled with left ventricular diastolic dysfunction, suggesting that 4D flow CMR serves as a sensitive noninvasive tool for early risk stratification in this population.

**Relevance statement:**

4D flow CMR identifies subclinical pulmonary hemodynamic alterations coupled with diastolic dysfunction in SLE patients, serving as a sensitive noninvasive tool for early risk stratification before irreversible vascular remodeling occurs.

**Key Points:**

- SLE patients without known pulmonary arterial hypertension show early pulmonary blood flow changes.
- 4D flow CMR detected asymmetric pulmonary flow redistribution in SLE patients.
- SLE patients exhibited altered left atrial function despite normal ventricles.
- Pulmonary flow changes correlated with left atrial remodeling in SLE.
- 4D flow CMR detects subclinical pulmonary hemodynamic differences in SLE.

**Graphical Abstract:**

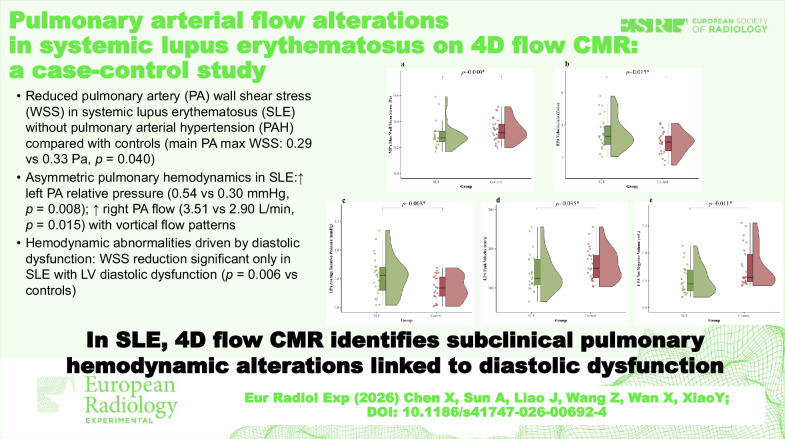

## Background

Pulmonary arterial hypertension (PAH) is a severe complication of systemic lupus erythematosus (SLE). While prevalence estimates vary widely based on diagnostic criteria, ranging from 0.5% to 17.5% in screening cohorts [1]. In Asian populations, SLE is the leading cause of connective tissue disease–associated PAH [2]. However, early detection remains difficult due to nonspecific symptoms and the insidious nature of disease onset [3].

Echocardiography and circulating biomarkers such as N-terminal pro-brain natriuretic peptide, NT‒proBNP, are widely used for screening, yet these conventional tools often miss early hemodynamic alterations and are limited in sensitivity [4]. More advanced techniques are needed to detect subclinical pulmonary vascular changes before irreversible remodeling occurs.

Four-dimensional (4D) flow cardiovascular magnetic resonance (CMR) is a noninvasive imaging modality that allows simultaneous quantification of key pulmonary artery flow metrics [5], including peak velocity, flow volume, wall shear stress (WSS), relative pressure, and vortex patterns [6]. In the context of SLE, chronic inflammation and immune complex-mediated vasculitis progressively impair endothelial function, leading to vascular stiffening and remodeling that precede the development of overt PAH [7]. Crucially, these early structural changes manifest as subtle hemodynamic disturbances detectable by 4D flow CMR. Specifically, markers such as lower WSS and energy loss serve as sensitive surrogates for diminished arterial compliance and inefficient flow transmission [8, 9]. This capability has been notably validated in systemic sclerosis, a connective tissue disease pathophysiologically similar to SLE. In systemic sclerosis, 4D flow CMR has successfully identified early pulmonary arterial alterations—characterized by abnormal helical flow and significantly lower WSS—even in patients without established PAH [10].

Despite advances in 4D flow CMR applications in systemic sclerosis-related pulmonary arterial assessment, analogous studies in SLE remain lacking [2]. To our knowledge, this is the first study to utilize 4D flow CMR in a dedicated cohort of SLE patients. Unlike previous research where SLE was examined only as an etiological subgroup of pulmonary hypertension [11], we focused on identifying subclinical hemodynamic alterations in patients without known PAH.

We hypothesized that SLE patients might exhibit distinct hemodynamic profiles in the pulmonary arteries compared to healthy controls, reflecting subclinical vascular involvement. Furthermore, higher left ventricular (LV) filling pressures associated with diastolic dysfunction can be transmitted backward to the pulmonary vasculature, potentially altering flow patterns [12]. Therefore, this study aims to characterize these hemodynamic differences using 4D flow CMR and to investigate their association with LV diastolic function.

## Materials and methods

### Ethics approval and consent to participate

The study was approved by the institutional review board, and all participants provided written informed consent in accordance with the Declaration of Helsinki prior to enrollment. The clinical protocol was reviewed and approved by the Clinical Research Ethics Committee of Shanghai Changzheng Hospital (Ethics Approval Number: 2023SL010).

### Study population

SLE patients without known PAH were recruited for this observational cross-sectional study from a single institution between August 4, 2023, and February 1, 2025. The diagnosis of SLE was confirmed according to the 2012 Systemic Lupus International Collaborating Clinics (SLICC) classification criteria [13].

**Fig. 1:**
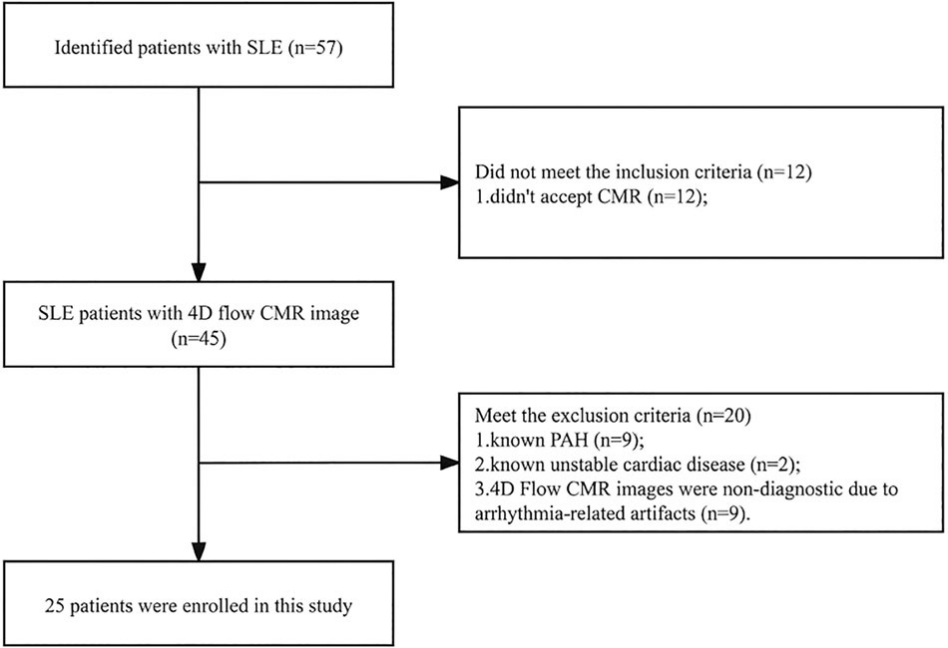
Patient selection process and application of inclusion/exclusion criteria. 4D, Four-dimensional; CMR, Cardiovascular magnetic resonance; PAH, Pulmonary arterial hypertension; SLE, Systemic lupus erythematosus

Detailed inclusion and exclusion criteria are summarized in Supplementary Table S1. Briefly, adults (≥ 18 years) with a confirmed diagnosis of SLE according to the 2012 SLICC [13] criteria were recruited. Patients with known pulmonary PAH, defined according to the 2015 European Society of Cardiology/European Respiratory Society guidelines [14], active infections, significant cardiac history, or contraindications to CMR were excluded. The inclusion and exclusion criteria are illustrated in the study flowchart (Fig. 1). A total of 57 patients were screened, of whom 25 met eligibility criteria and were enrolled.

A group of age-matched healthy volunteers without known cardiovascular or autoimmune disease served as the control group.

This observational cross-sectional study was conducted and reported in accordance with the STROBE (Strengthening the Reporting of Observational Studies in Epidemiology) guidelines.

### CMR protocol

All CMR scans were performed on a 3-T scanner (SIGNA™ Premier; GE Healthcare). Imaging was performed with retrospective electrocardiographic gating and a navigator for respiratory gating. Long-axis 2-chamber, 4-chamber, and LV outflow tract cine images were acquired using a segmented balanced steady-state free precession‒bSSFP sequence. Imaging parameters were repetition time/echo time = 6.5 ms/3.0 ms, field of view = 420 × 340 mm², matrix = 160 × 160, slice thickness = 8 mm, slice gap = 2 mm, and 10 slices per stack.

The imaging protocol included a 4D flow sequence with volumetric coverage of the right and LV outflow tracts and the pulmonary arteries. The average acquisition time for the 4D flow sequence was approximately 8–12 min, depending on the heart rate and respiratory gating efficiency. Key acquisition parameters are summarized in Table 1.

**Table 1:** CMR acquisition parameters for 4D flow imaging

Parameter	Value
FOV, mm	360 × 288 × 90
Acquired voxel size, mm	2.4 × 2.4 × 4.0
Reconstructed voxel size, mm	1.4 × 1.4 × 2.0
Flip angle, degrees	15
TE, ms	2.1
TR, ms	3.8
VENC, cm/s	150–200
(Reconstructed) cardiac phases	20

*CMR* Cardiovascular magnetic resonance, *FOV* Field of view, *TE* Echo time, *TFE* Turbo field-echo, *TR* Repetition time, *VENC* Velocity encoding

### Image analysis

4D flow data were analyzed using a commercial software platform (CVI42 v5.11, Circle Cardiovascular Imaging). Manual segmentation of the pulmonary artery tree was performed, including the main pulmonary artery (MPA), right pulmonary artery (RPA), and left pulmonary artery (LPA).

**Fig. 2:**
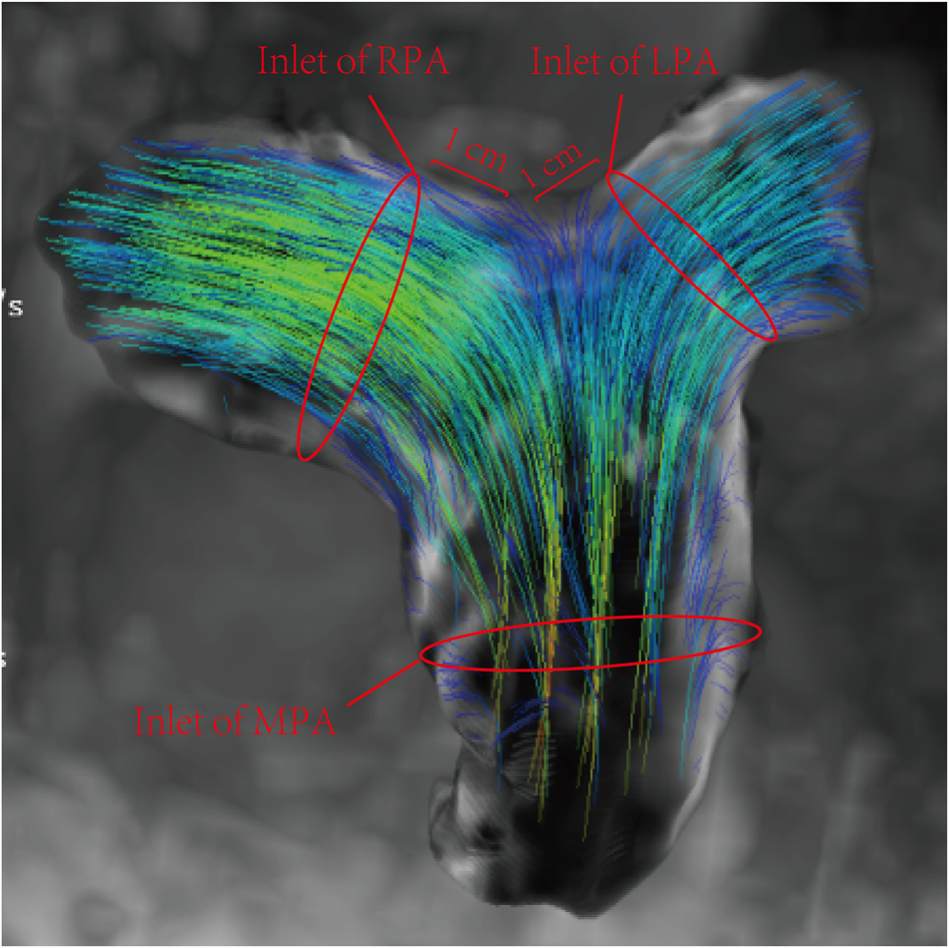
Positioning of analysis planes in the pulmonary arteries for 4D flow analysis. 4D, Four-dimensional; LPA, Left pulmonary artery; MPA, Main pulmonary artery; RPA, Right pulmonary artery

According to previous research [15], cross-sectional analysis planes were placed at three locations: (1) the MPA inlet (at the level of the pulmonary valve), (2) 1 cm distal to the origin of the LPA, and (3) 1 cm distal to the origin of the RPA, as shown in Fig. 2. From these planes, flow-derived parameters were calculated, including forward volume, negative volume, net flow volume, peak velocity, maximum flow rate, average relative pressure, and WSS. Energy loss was quantified as the rate of energy dissipation due to viscous forces within the pulmonary arterial volume. It was calculated using the CVI42 software based on the viscous dissipation function described by Barker et al [9]. The software integrates the sum of the squared spatial velocity gradients over the segmented volume.

Bi-ventricular and Atrial Function were measured from cine images using Simpson’s biplane method. Ventricular and atrial volumes were indexed to body surface area calculated using the Mosteller formula [16].

All quantitative measurements were independently performed by two experienced radiologists, and final values were obtained by averaging the two readings to enhance measurement reliability.

### Echocardiographic assessment and diastolic function

All SLE patients underwent echocardiography within a week of the CMR scan. Left ventricular diastolic function was evaluated according to the 2016 American Society of Echocardiography/European Association of Cardiovascular Imaging. Guidelines for the Evaluation of Left Ventricular Diastolic Function [17]. For patients with LV ejection fraction preserved (≥ 50%), diastolic dysfunction was identified based on four recommended variables: (1) average E/e’ > 14; (2) septal e’ velocity < 7 cm/s or lateral e’ velocity < 10 cm/s; (3) tricuspid regurgitation velocity > 2.8 m/s; and (4) left atrial (LA) volume index > 34 mL/m².

Patients were classified into two subgroups based on these criteria:

- Group 0 (no diastolic dysfunction) = meeting < 50% of the pathological cutoffs;
- Group 1 (diastolic dysfunction) = meeting ≥ 50% of the pathological cutoffs.

### Statistical analysis

Statistical analyses and data visualization were performed using R statistical software (version 4.4.3; R Foundation for Statistical Computing, Vienna, Austria). Continuous variables were analyzed using the base stats package. Data visualization was performed using the ggplot2 package. Between-group comparisons were visualized and annotated with significance levels using the ggpubr package. The correlation heatmap was generated using the corrplot package. Continuous variables were assessed for normality with the Shapiro–Wilk test and are reported as mean ± standard deviation or median (interquartile range) as appropriate. Between-group comparisons were made using independent-samples *t*-tests for normally distributed data or Wilcoxon rank-sum tests for nonnormal data. Spearman’s rank correlation was used to assess associations between 4D flow parameters and clinical or laboratory variables. A two-tailed *p*-value < 0.05 was considered statistically significant. Gemini 2.5 (Google) was used solely for grammar checking and language polishing. All text was reviewed and edited by the authors, who take full responsibility for the entire content of the manuscript. No figures, tables, images, or videos were generated or modified using generative AI.

## Results

### Participant characteristics

Twenty-five SLE patients without known PAH and 25 healthy controls were included. The SLE group comprised 18 females and 7 males, compared to 14 females and 11 males in the control group (*p* = 0.035). No significant differences were observed in age (*p* = 0.475) or body mass index. Consistent with the exclusion criteria, none of the included SLE patients had a history of documented coronary artery disease, heart failure, valvular heart disease, or severe arrhythmia. Regarding cardiovascular risk factors in the SLE cohort, 4 patients (16%) had hypertension, 7 patients (28%) had hyperlipidemia, and 1 patient (4%) was a current smoker.

Several hematologic differences were noted. The SLE group had significantly lower erythrocyte count, hematocrit, hemoglobin, platelet count, and higher red cell distribution width compared to controls. Additional demographic and laboratory findings are summarized in Table 2.

**Table 2:** Baseline demographic and laboratory characteristics of SLE patients and controls

	SLE group	Control group	*p*-value
Sex (F/M)	18/7	14/11	0.035*
Age (years)	34.24 ± 13.25	38.30 ± 16.31	0.475
Body mass index (kg/m²)	22.00 ± 3.38	23.29 ± 3.05	0.175
Neutrophils (%)	64.05 ± 12.86	59.64 ± 9.54	0.194
Aspartate aminotransferase (U/L)	24.28 ± 11.34	25.20 ± 7.27	0.252
Creatinine (mg/dL)	62.17 ± 13.27	63.30 ± 14.14	0.501
Red cell distribution width (%)	13.98 ± 2.04	13.22 ± 2.02	0.046*
Leukocyte count (× 10⁹/L)	5.35 ± 3.13	6.71 ± 2.07	0.019*
Lymphocytes (%)	25.13 ± 11.13	31.18 ± 8.83	0.048*
Platelet count (× 10⁹/L)	198.36 ± 89.86	247.50 ± 63.88	0.038*
Hemoglobin (g/dL)	114.12 ± 20.13	137.55 ± 18.25	0.002*
Total protein (g/dL)	63.02 ± 13.95	72.28 ± 6.64	0.005*
Albumin-globulin ratio	1.28 ± 0.37	1.52 ± 0.31	0.022*
Total bilirubin (mg/dL)	8.75 ± 4.36	13.98 ± 5.98	<0.001*
Indirect bilirubin (mg/dL)	7.37 ± 4.32	13.35 ± 6.04	<0.001*

Data presented as mean ± standard deviation

*p* < 0.05; statistically significant values are indicated in bold and denoted by an asterisk(*)

### 4D flow-derived pulmonary hemodynamic parameters

**Fig. 3:**
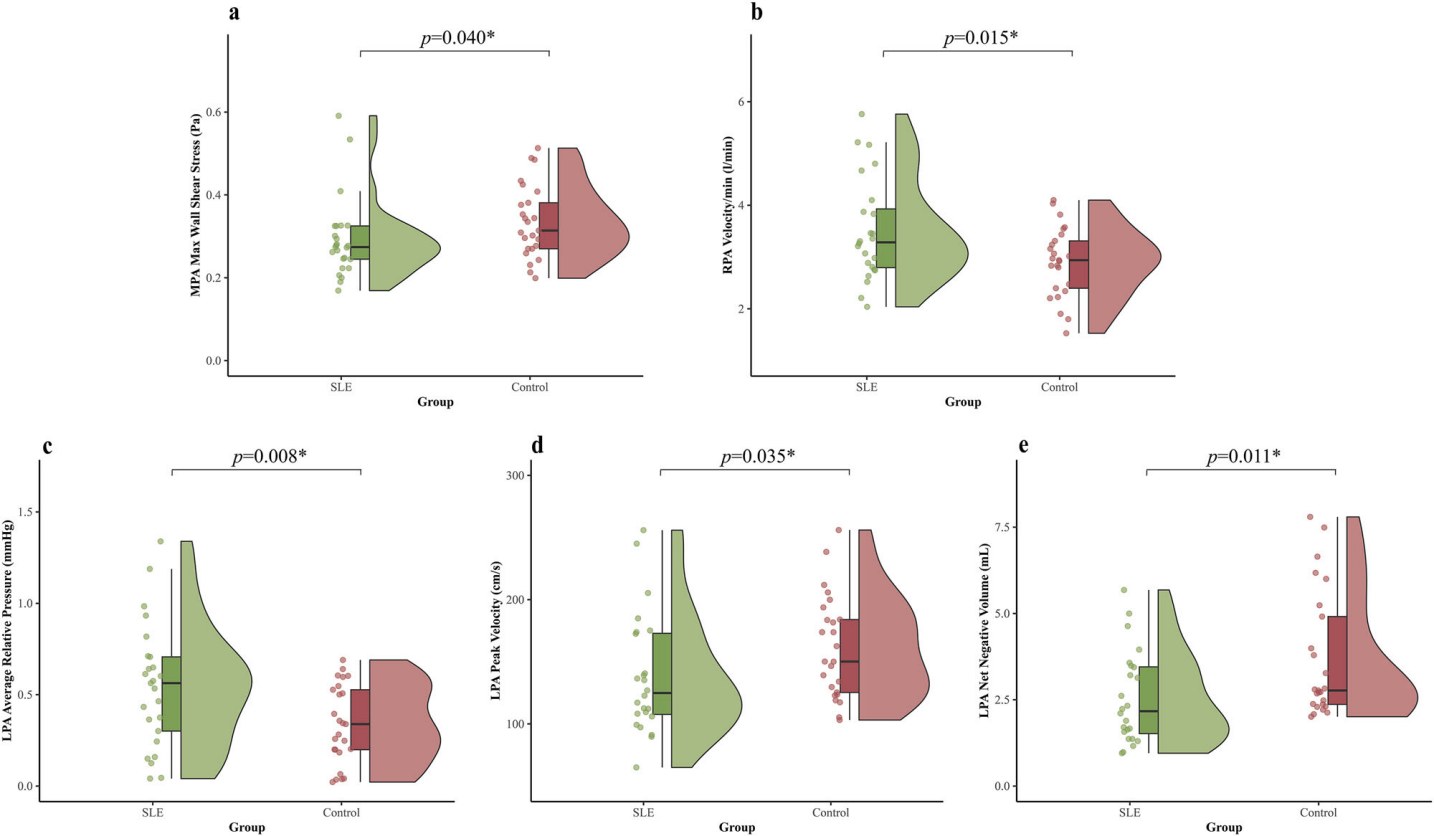
Comparison of key 4D flow hemodynamic parameters between SLE patients and healthy controls. **a** Maximum WSS in the MPA. **b** Mean flow rate in the RPA. **c** Average relative pressure in the LPA. **d** Peak velocity in the LPA. **e** Net reverse flow volume in the LPA. Statistical comparisons were performed using *t*-tests or Wilcoxon tests as appropriate; *p*-values are indicated above each pair; * *p* < 0.05. 4D, Four-dimensional; LPA, Left pulmonary artery; MPA, Main pulmonary artery; RPA, Right pulmonary artery; SLE, Systemic lupus erythematosus; WSS, Wall shear stress

In Table 3 and Fig. 3, we observed significant differences in pulmonary artery hemodynamics between SLE patients and controls. In the MPA, the maximum WSS was lower in SLE patients (0.29 ± 0.10 (mean ± standard deviation) *versus* 0.33 ± 0.09 Pa, *p* = 0.040). In contrast, velocity/min in the RPA was higher in SLE than in controls (3.51 ± 0.99 *versus* 2.90 ± 0.67 L/min, *p* = 0.015). The LPA in SLE showed multiple alterations: average relative pressure was higher (0.54 ± 0.34 *versus* 0.30 ± 0.26 mmHg, *p* = 0.008); however, net negative volume and peak velocity were lower compared to controls (2.54 ± 1.34 *versus* 3.68 ± 1.82 mL and 138.43 ± 48.37 *versus* 161.27 ± 41.27 cm/s, *p* = 0.011 and 0.035).

**Table 3:** SLE *versus* controls: key 4D flow hemodynamic parameters in the pulmonary arteries

	SLE group	Control group	*p*-value
MPA max wall shear stress (Pa)	0.29 ± 0.10	0.33 ± 0.09	0.040*
RPA velocity/min (L/min)	3.51 ± 0.99	2.90 ± 0.67	0.015*
LPA average relative pressure (mmHg)	0.54 ± 0.34	0.30 ± 0.26	0.008*
LPA peak velocity (cm/s)	138.43 ± 48.37	161.27 ± 41.27	0.035*
LPA net negative volume (mL)	2.54 ± 1.34	3.68 ± 1.82	0.011*

*LPA* Left pulmonary artery, *MPA* Main pulmonary artery, *RPA* Right pulmonary artery, *SLE* Systemic lupus erythematosus

*p* < 0.05; statistically significant values are indicated in bold and denoted by an asterisk(*)

**Fig. 4:**
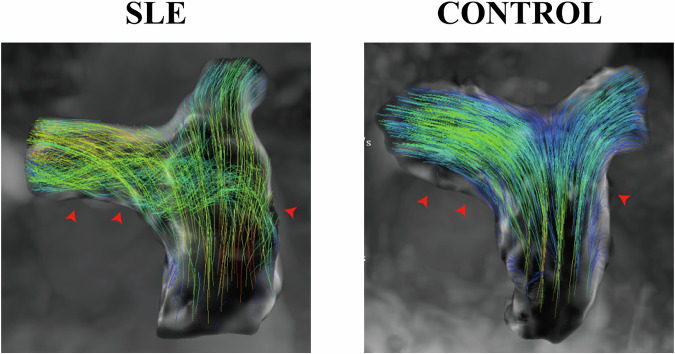
Representative pulmonary artery 4D flow images from a patient with SLE (left) and a healthy control (right). These streamline visualizations depict blood flow patterns and velocities in the main and branch pulmonary arteries. 4D, Four-dimensional; SLE, Systemic lupus erythematosus

Qualitative analysis of streamline visualization revealed the presence of vortical flow patterns in the RPA of SLE patients (Fig. 4), contrasting with the uniform, laminar flow observed in healthy controls.

To validate the flow measurements, we compared the net forward volume in the MPA derived from 4D flow CMR with the right ventricular stroke volume obtained from cine imaging. The analysis demonstrated a strong positive correlation between the two methods (*r* = 0.795, *p* < 0.0001), confirming the internal consistency of the hemodynamic data.

### Impact of LV diastolic function on pulmonary hemodynamics

To investigate the association between cardiac functional impairment and pulmonary hemodynamics, SLE patients were stratified based on echocardiographic assessment of diastolic function. The cohort was divided into patients with normal diastolic function (Group 0, *n* = 13) and those with diastolic dysfunction (Group 1, *n* = 12).

**Fig. 5:**
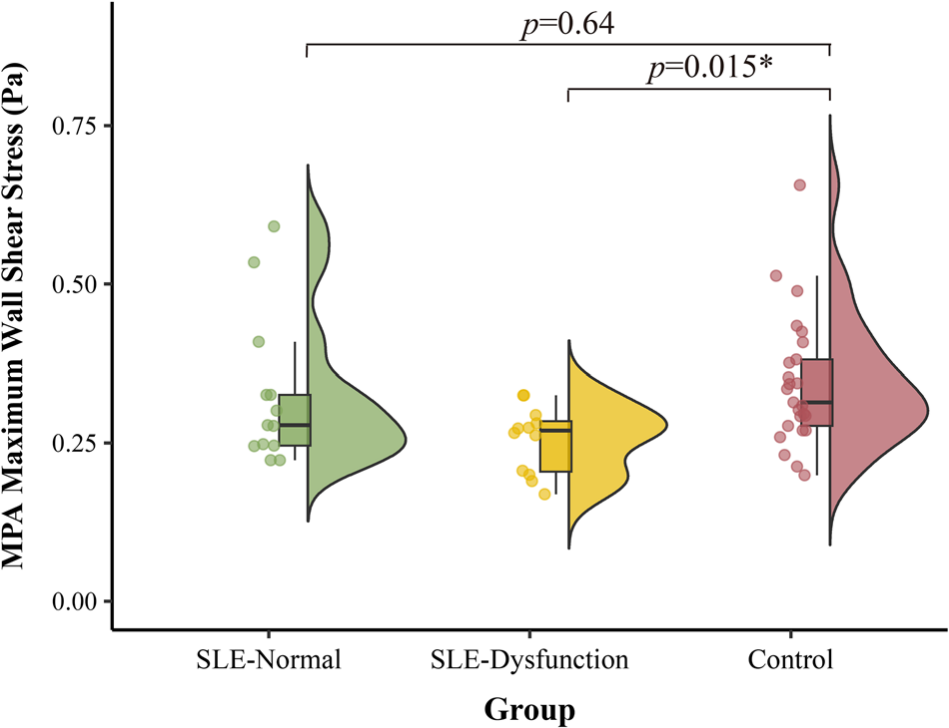
Impact of diastolic function on the main pulmonary artery WSS. Comparison of maximum WSS in the MPA among SLE patients with normal diastolic function (SLE-Normal), SLE patients with diastolic dysfunction (SLE-Dysfunction), and healthy controls. MPA, Main pulmonary artery; SLE, Systemic lupus erythematosus; WSS, Wall shear stress

Subgroup analysis revealed that the reduction in WSS observed in the overall SLE cohort was predominantly driven by the subgroup with diastolic dysfunction. Specifically, Group 1 exhibited significantly lower maximum WSS in the MPA compared to healthy controls (0.255 ± 0.052 *versus* 0.343 ± 0.103 Pa, *p* = 0.006), whereas Group 0 showed no statistically significant difference compared to controls (0.325 ± 0.118 *versus* 0.343 ± 0.103 Pa, *p* = 0.332). A similar trend was observed in the LPA, where maximum WSS was significantly lower in Group 1 compared to controls (*p* = 0.048), but preserved in Group 0 (*p* = 0.373). Additionally, Group 1 demonstrated a significantly higher MPA regurgitation fraction compared to Group 0 (*p* = 0.030). These findings are illustrated in Fig. 5.

### Bi-ventricular and atrial function

Quantitative assessment of bi-atrial and bi-ventricular parameters revealed that most conventional volumetric indices—such as end-diastolic volume, end-systolic volume, and ejection fraction—did not differ significantly between SLE and control groups.

Left atrial volumetric analysis indexed to body surface area revealed distinct functional alterations. While the maximum LA volume index did not differ significantly between groups (24.89 ± 9.61 *versus* 27.26 ± 8.32 mL/m², *p* = 0.355), the minimum LA volume index was significantly lower in SLE patients (8.73 ± 5.46 *versus* 11.42 ± 5.59 mL/m², *p* = 0.039). Consequently, LA ejection fraction was significantly higher in the SLE group (67.87 ± 9.40 *versus* 62.90 ± 13.62%, *p* = 0.023), suggestive of a hyperdynamic compensatory state rather than structural enlargement. Key atrial parameters are summarized in Table 4. Additional structural indices are summarized in Supplementary Table S1.

**Table 4:** Left atrial and ventricular structural and functional parameters

	SLE group	Control group	*p*-value
LAVI max (mL/m²)	24.89 ± 9.61	27.26 ± 8.32	0.355
LAVI min (mL/m²)	8.73 ± 5.46	11.42 ± 5.59	0.039*
LA total EF (%)	67.87 ± 9.40	62.90 ± 13.62	0.023*

*LA* Left atrium, *LAEF* Left atrial ejection fraction

*p* < 0.05; statistically significant values are indicated in bold and denoted by an asterisk(*)

### Correlation analysis

**Fig. 6:**
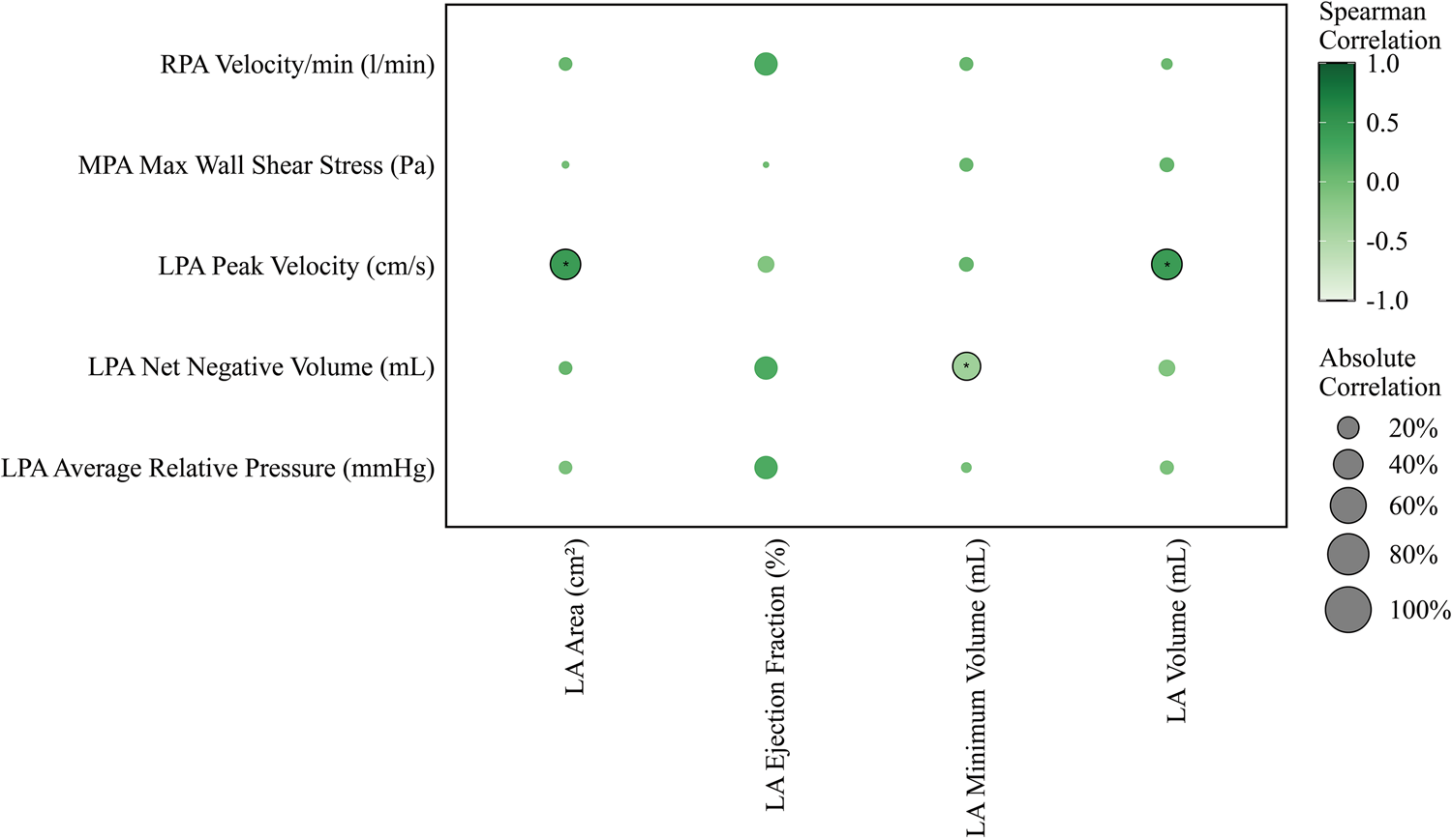
Heatmap of Spearman correlation coefficients between 4D flow pulmonary parameters and LA markers. Darker colors indicate stronger correlations. Significant correlations are marked with an asterisk (*). LA, Left atrium; LPA, Left pulmonary artery; MPA, Main pulmonary artery; RPA, Right pulmonary artery; SLE, Systemic lupus erythematosus

Significant correlations were observed among 4D flow metrics and LA parameters. The results were demonstrated in Fig. 6. Several 4D flow variables were associated with LA morphology. LPA peak velocity correlated positively with LA volume (ρ = 0.34, *p* = 0.016) and LA area (ρ = 0.28, *p* = 0.038). LPA net negative volume was inversely correlated with LA minimum volume (ρ = -0.31, *p* = 0.032).

## Discussion

In this study, we identified distinct pulmonary hemodynamic alterations in SLE patients without known PAH, characterized by significantly lower WSS in the main pulmonary artery and asymmetric flow redistribution. To our knowledge, this is the first investigation to utilize 4D flow CMR in a dedicated SLE cohort. Specifically, we quantified these subclinical vascular patterns and, crucially, demonstrated their association with LV diastolic dysfunction. These findings suggest that pulmonary vascular alterations in SLE are not isolated events but are intimately coupled with LV diastolic function.

### Main pulmonary artery hemodynamics

The hemodynamic alterations observed in our SLE cohort—specifically the lowered WSS and the emergence of vortical flow—closely resemble the preclinical vascular phenotypes described by previous investigators in other connective tissue diseases. Specifically, the lower WSS observed in our SLE cohort mirrors the hemodynamic phenotype reported by Ikoma et al [10] in patients with systemic sclerosis without overt PAH, and resembles the flow alterations described by Tang et al [8] in established pulmonary arterial hypertension. This consistency across different connective tissue diseases suggests that low WSS may represent a shared subclinical marker of pulmonary vascular involvement.

Furthermore, the vortical flow patterns we identified (Fig. 4) align with previous observations that flow instability often manifests as a sensitive marker of higher pulmonary vascular resistance [18]. Thus, our findings likely represent the SLE-specific manifestation of this generalized subclinical vasculopathy.

### Pathophysiological mechanisms

Regarding the potential drivers of these hemodynamic alterations, our data support a multifactorial mechanism. While SLE-associated chronic inflammation and immune complex deposition likely contribute to intrinsic endothelial dysfunction and vascular stiffening [7], our subgroup analysis highlights the pivotal role of cardiopulmonary hemodynamic coupling. The observation that maximum MPA WSS was significantly lower in patients with diastolic dysfunction compared to healthy controls (*p* = 0.006) strongly suggests that higher LV filling pressures are passively transmitted backward to the pulmonary vasculature [12]. According to the paradigm of PAH associated with left heart disease, this chronic retrograde pressure transmission reduces pulmonary arterial compliance and increases pulsatile afterload [12]. The “low WSS” state we observed likely reflects this diminished arterial wall compliance and the resultant blunting of near-wall velocities, a phenomenon similarly described in computational models of PAH [8]. Therefore, we propose that in a subset of SLE patients, the pulmonary vascular profile is a composite result of systemic inflammatory vasculopathy and hemodynamic stress transmitted from the left heart, consistent with the pathophysiology of “combined pre- and post-capillary” involvement often seen in complex connective tissue diseases [12].

### Asymmetric flow redistribution

We observed a distinct pattern of hemodynamic asymmetry between the left and right pulmonary arteries. Specifically, the LPA exhibited higher relative pressure alongside lower peak velocity and net reverse flow. These combined findings point to increased downstream impedance and potential geometric distortion, which can induce secondary flows and dampen forward momentum [19]. In response to this LPA constraint, blood flow appears to be compensatorily shunted toward the RPA. While RPA velocity was higher, the concurrent presence of disturbed vortical patterns indicates significant energy dissipation and lower hydraulic efficiency—a phenomenon conceptually analogous to the flow inefficiencies described in Fontan circulation [20]. This maladaptive redistribution mirrors the regional flow heterogeneity characteristic of pulmonary arterial hypertension [21], a pattern which has been shown to be detectable by 4D flow CMR even before the onset of overt pressure elevation [10]. This underscores the superior sensitivity of 4D flow CMR in detecting occult vascular alterations compared to conventional echocardiography [22].

### Coupling of pulmonary hemodynamics and LA function

Despite the absence of structural enlargement (no difference in LAVI max), SLE patients exhibited significantly lower LAVI min and a correspondingly higher LA ejection fraction, indicating a hyperdynamic LA profile. Such a pattern is consistent with early functional remodeling described in diastolic dysfunction, where minimal LA volume and phasic emptying are highly sensitive markers of subtle hemodynamic alterations [23]. Given the pulmonary vascular alterations we identified—including lower MPA WSS, vortical flow, and higher LPA impedance—these findings suggest that alterations in pulmonary arterial compliance and right-to-left ventricular interdependence may modestly modify LV relaxation and transmitral filling. This effectively increases the functional load on the left atrium even in the absence of overt chamber dilation [24].

This interpretation aligns with our correlation analysis showing that LPA peak velocity was positively associated with LA volume and area. Such associations indicate that pulmonary artery flow characteristics and LA emptying are not independent phenomena, but instead reflect a coupled cardiopulmonary system in which subclinical pulmonary vascular changes and early LV diastolic impairment converge to shape atrial phasic behavior.

### Clinical implications

Current echocardiographic screening often lacks sensitivity for early-stage pulmonary vascular involvement. Our findings suggest that 4D flow CMR could serve as a vital noninvasive risk stratification tool, particularly for SLE patients with echocardiographic diastolic dysfunction. We propose that patients with Grade I or higher diastolic dysfunction—even with normal estimated pulmonary pressures—may benefit from 4D flow assessment. Acting as a “gatekeeper,” 4D flow CMR can detect occult hemodynamic markers such as low MPA WSS or flow asymmetry, thereby identifying candidates for closer monitoring or early specialized referral before irreversible remodeling occurs.

### Limitations

Our study has several limitations. First, this was a single-center cross-sectional study with a relatively small sample size. Consequently, we cannot establish a definitive causal link between these early flow alterations and the future progression to overt PAH. Second, invasive right heart catheterization and late gadolinium enhancement (LGE) were not performed. These procedures require invasive instrumentation or contrast agent administration, which were ethically unjustified for our asymptomatic patients and healthy controls. Therefore, we could not validate CMR-derived metrics against invasive pressures or assess myocardial fibrosis. Finally, standardized normal reference values for pulmonary 4D flow parameters have not yet been established. As a result, we cannot determine whether the observed values represent absolute pathological anomalies, but rather statistically significant differences compared to the control group.

### Conclusions

This study demonstrates that SLE patients without overt pulmonary hypertension exhibit subclinical hemodynamic alterations, specifically lower wall shear stress and asymmetric flow redistribution. These changes are closely coupled with LV diastolic dysfunction and subtle LA remodeling. 4D flow CMR effectively detects these early vascular alterations, highlighting its potential as a sensitive, noninvasive tool for early risk stratification and monitoring in SLE patients.

## Supplementary information

**Additional file 1:** **Table S1**. Inclusion and exclusion criteria for the study population. **Table S2**. Structural parameters of cardiac chambers in SLE patients.

## Data Availability

The datasets generated and analyzed during the current study are available from the corresponding author upon reasonable request.

## Abbreviations

4D: Four-dimensional
CMR: Cardiovascular magnetic resonance
LA: Left atrial
LPA: Left pulmonary artery
LV: Left ventricular
MPA: Main pulmonary artery
PAH: Pulmonary arterial hypertension
RPA: Right pulmonary artery
SLE: Systemic lupus erythematosus
SLICC: Systemic Lupus International Collaborating Clinics
WSS: Wall shear stress
