# Supplementary material for: Pulmonary arterial flow alterations in systemic lupus erythematosus on 4D flow CMR: a case-control study
Source: Eur Radiol Exp. 2026 Mar 4;10:25. doi: 10.1186/s41747-026-00692-4 (PMC12961025; doi:10.1186/s41747-026-00692-4)
Supplement: Supplementary file 1 — Additional file 1: Table S1. Inclusion and exclusion criteria for the study population. Table S2. Structural parameters of cardiac chambers in SLE patients. [file 41747_2026_692_MOESM1_ESM.pdf]

Pulmonary arterial flow alterations in systemic lupus erythematosus on 4D flow CMR: a case-control study

ELECTRONIC SUPPLEMENTARY MATERIAL

Table S1 Inclusion and exclusion criteria for the study population

Numbers in brackets refer to the reference list in the main text. *CMR* Cardiovascular magnetic resonance, ERS European Respiratory Society, *ESC* European Society of Cardiology, *MRI* magnetic resonance imaging, *PAH* pulmonary arterial hypertension, *PAP* Pulmonary arterial pressure, *SLE* Systemic lupus erythematosus, *SLICC* Systemic Lupus International Collaborating Clinics, *TRV* peak tricuspid regurgitation velocity.

Inclusion Criteria	Exclusion Criteria
1. **Age ≥ 18 years**<br>2. Confirmed Diagnosis**:** SLE according to the 2012 SLICC classification criteria [11].<br>3. Imaging Safety**:** No contraindications to MRI (e.g., non-MRI-conditional implants, severe claustrophobia).	**1.** active infection **at the time of enrollment.**<br>2. Pulmonary Vascular Disease**:** Known PAH, defined as TRV ≥ 3.0 m/s on echocardiography (estimated systolic PAP ≥ 35 mmHg), per 2015 ESC/ERS guidelines [12].<br>3. Comorbidities**:** Known malignancy, unstable cardiac disease, history of cardiac surgery, or severe arrhythmia.<br>4. Medication/Safety**:** Severe allergic reactions to biological therapies or receipt of live-attenuated vaccines within the past 4 weeks.<br>5. Physiological State**:** Current pregnancy or lactation.<br>6. Image Quality**:** Severe artifacts on 4D Flow CMR images precluding analysis.

Table S2. Structural parameters of cardiac chambers in SLE patients

	SLE	Control	P-value
**Left Ventricle**			
**Left Ventricular Ejection Fraction**	62.76	63.31	0.819
**Left Ventricular End-Diastolic Volume Index**	76.79	76.26	0.954
**Left Ventricular End-Systolic Volume Index**	28.86	27.88	0.614
**Left Ventricular Stroke Volume Index**	47.93	48.37	0.641
**Right Ventricle**			
**Right Ventricular Ejection Fraction**	55.72	56.8	0.526
**Right Ventricular End-Diastolic Volume Index**	73.24	73.37	0.831
**Right Ventricular End-Systolic Volume Index**	35.80	31.32	0.485
**Right Ventricular Stroke Volume Index**	42.68	42.44	0.938
**Left Atrium**			
**Left Atrial Ejection Fraction**	67.87	61.90	0.023
**Left Atrial End-Diastolic Volume Index**	9.97	12.23	0.046
**Left Atrial End-Systolic Volume Index**	22.33	24.73	0.187
**Maximum Left Atrial Volume Index**	24.89	27.26	0.355
**Minimum Left Atrial Volume Index**	8.73	11.42	0.039
**Right Atrium**			
**Right Atrial Ejection Fraction**	52.81	56.12	0.642
**Right Atrial End-Diastolic Volume Index**	15.60	14.44	0.393
**Right Atrial End-Systolic Volume Index**	29.47	28.45	0.548
**Maximum Right Atrial Volume Index**	30.84	29.65	0.509
**Minimum Right Atrial Volume Index**	14.47	13.15	0.485

Structural indices of left and right atria and ventricles, as quantified by cardiac magnetic resonance. All parameters were indexed to body surface area (BSA) calculated using the Mosteller formula.
